# *Cronobacter* spp. in Commercial Powdered Infant Formula Collected From Nine Provinces in China: Prevalence, Genotype, Biofilm Formation, and Antibiotic Susceptibility

**DOI:** 10.3389/fmicb.2022.900690

**Published:** 2022-05-27

**Authors:** Peng Fei, He Jing, Yan Ma, Gege Dong, Yunhe Chang, Zhaoxu Meng, Shilong Jiang, Qinggang Xie, Shuzhen Li, Xi Chen, Weiwei Yang

**Affiliations:** ^1^School of Zhang Zhongjing Health Care and Food, Nanyang Institute of Technology, Nanyang, China; ^2^Food and Pharmaceutical Engineering Institute, Guiyang University, Guiyang, China; ^3^Inner Mongolia Meng Niu Dairy Industry (Group) Co. Ltd. R&D Center, Hohhot, China; ^4^Heilongjiang Feihe Dairy Co., Ltd., Beijing, China; ^5^Department of Immunology, College of Basic Medical Sciences, Shenyang Medical College, Shenyang, China; ^6^Institute of Integrated Agricultural Development Research, Guizhou Academy of Agriculrural Sciences, Guiyang, China; ^7^The Department of Food Science, Shenyang Medical College, Shenyang Medical College, Shenyang, China

**Keywords:** *Cronobacter* spp., commercial powdered infant formula, genotype, biofilm formation, antibiotic susceptibility

## Abstract

The purpose of this study was to investigate the prevalence of *Cronobacter* spp. in commercial powdered infant formula (PIF) from nine provinces in China from March 2018 to September 2020, and to reveal the genotype, biofilm-forming ability, and antibiotic susceptibility of these isolates. A total of 27 *Cronobacter* strains, consisting of 22 *Cronobacter sakazakii* strains, 3 *Cronobacter malonaticus* strains, 1 *Cronobacter turicensis* strain, and 1 *Cronobacter dublinensis* strain, were isolated from 3,600 commercial PIF samples with a prevalence rate of 0.75%. Compared with the other 8 provinces, PIF from Shaanxi province had a higher prevalence rate (1.25%) of *Cronobacter* spp. These isolates were divided into 14 sequence types (STs), and 6 *Cronobacter* serotypes. The main *Cronobacter* STs were ST4, ST1, and ST64, and the dominant *Cronobacter* serotype was *C. sakazakii* serotype O2. Approximately 88.89% of *Cronobacter* isolates had a strong ability (OD_595_ > 1) to form biofilms on tinplate, among which the strains with ST4 were more dominant. All isolates were susceptible to ampicillin-sulbactam, ceftriaxone, cefotaxime, sulfadiazine, sulfadoxine, trimethoprim-sulfamethoxazole, gentamicin, tetracycline, ciprofloxacin, and colistin, while 55.56 and 96.30% isolates were resistant to cephalothin and vancomycin, respectively. Taken together, our findings highlighted the contamination status and characterization of *Cronobacter* spp. in commercial PIF from nine provinces of China, and provided guidance for the effective prevention and control of this pathogen in the production of PIF.

## Introduction

*Cronobacter* spp. is an important opportunistic food-borne pathogen and can infect people of all ages (Sonbol et al., 2013; Kadlicekova et al., 2018). Due to lower immunity, newborns, especially low-birth-weight infants, are more likely to be infected by *Cronobacter* spp. compared to other groups of people, and suffer from bacteremia, necrotizing enterocolitis, meningitis, and even death (Caubilla-Barron et al., 2007; Fei et al., 2015). Currently, ingestion of powdered infant formula (PIF) contaminated with *Cronobacter* spp. is the leading cause of infection in newborns (Joseph and Forsythe, 2011; Yan et al., 2012). Therefore, the prevalence of *Cronobacter* spp. in commercial PIF has been a constant concern.

Genotyping of *Cronobacter* spp. isolated from PIF can reveal its molecular characterization, and contribute to the prevention and source tracking of this pathogen (Fei et al., 2018; Ling et al., 2021). Multilocus sequence typing (MLST) is considered a powerful tool for detection and genotyping of *Cronobacter* spp., because of discrimination at species level and information sharing platform (Joseph and Forsythe, 2012; Joseph et al., 2012; Li C. et al., 2020; Ling et al., 2021). To date, 3,506 *Cronobacter* spp. isolates have been classified into 794 sequence types (STs) using MLST method according to the records of *Cronobacter* PubMLST database (http://www.pubmlst.org/cronobacter). In addition, O-antigen serotyping based on lipopolysaccharide structure associated with the proinflammatory response of host to infection should also be implemented to increase the understanding of *Cronobacter* spp. for epidemiological purposes (Blazkova et al., 2015; Fei et al., 2017b).

Some studies have shown that secondary contamination during packaging and transportation is the main cause of *Cronobacter* spp. contamination in PIF, and the strong ability of *Cronobacter* spp. to form biofilms increases this possibility (Lee et al., 2012; Yang et al., 2013; Brandl et al., 2014). On the one hand, biofilm helps *Cronobacter* spp. firmly adhere to the surface of equipment and packaging materials, increasing the possibility of contamination of PIF with this pathogen (Aly et al., 2019). On the other hand, the existence of biofilm reduces the bactericidal effect of disinfectants and plays a protective role effect on *Cronobacter* spp. (Lehner et al., 2005). Therefore, the biofilm-forming ability of *Cronobacter* spp. isolated from PIF should be evaluated, which can contribute to the development of effective prevention and control measures of this pathogen.

In addition, the monitoring of antibiotic resistance of *Cronobacter* spp. is also necessary in order to update the effective antibiotic treatment regimen (Li Y. et al., 2020). For now, antibiotics are still the most effective therapeutic method for human *Cronobacter* infection (Fei et al., 2017a). However, long-term use of antibiotics or horizontal gene transfer are likely to result in increased antibiotic resistance in *Cronobacter* spp. (Holy et al., 2019). More importantly, the occurrence of multiple-drug-resistant *Cronobacter* strains can lead to the failure of antibiotics treatment, thereby increasing the epidemiological risk of this pathogen (Zeng et al., 2018; Li C. et al., 2020).

From the above considerations, we investigated the contamination by *Cronobacter* spp. in commercial PIF from supermarkets in nine provinces in China from March 2018 to September 2020. A total of 27 *Cronobacter* spp. isolates were detected, and genotyped using multilocus sequence typing (MLST) and O-antigen serotyping. Besides, the ability of biofilms formation on tinplate and antibiotic susceptibility of all isolates were tested.

## Materials and Methods

### Sampling

From March 2018 to September 2020, a total of 3,600 commercial PIF samples were randomly purchased from supermarkets in nine provinces of China, including Heilongjiang (*n* = 400), Xinjiang (*n* = 400), Jilin (*n* = 400), Hebei (*n* = 400), Henan (*n* = 400), Shaanxi (*n* = 400), Guizhou (*n* = 400), Yunnan (*n* = 400), and Fujian (*n* = 400). As shown in the Supplementary Figure 1, the nine provinces were distributed in the northeast, northwest, central, southwest, and southeast of China. The sample details are given in Supplementary Table 1, including collection region, collection time, manufacturers, place of origin, and raw milk types. Besides, all PIF samples are from local enterprises in China. After purchase, the packaging surfaces of all samples were sterilized with 75% alcohol, placed in sterile convenient insulated containers with ice packs, and quickly transferred to the laboratory.

### Isolation and Identification of *Cronobacter* spp.

The suspected *Cronobacter* spp. were isolated from commercial PIF samples, and identified at the biochemical level according to the method as mentioned in the national food safety standard method for food microbiological examination as used in China GB4789.40-2010 (Ministry of Health of the People's Republic of China, 2010). Briefly, 100 g of PIF was fully dissolved in 900 ml sterile buffered peptone water followed by incubation at 36°C for 18 h. One milliliter culture was selectively enriched in 10 ml modified lauryl sulfate tryptose broth-vancomycin (mLST-Vm) medium with incubation at 44°C for 24 h. The above bacterial solution was spread on the Druggan-Forsythe-Iversen (DIF) medium, and the blue-green colonies were identified as the suspected colonies. All typical colonies were inoculated into tryptic soy broth, followed by cultivation at 37°C for 18 h. Then, genomic DNA of the culture was extracted using TIANamp Bacterial DNA Kit [Tiangen Biotech (Beijing) Co, Ltd, Beijing, China]. Finally, species-level identification of the isolates was performed using *fusA* gene sequencing, as previously reported (Fei et al., 2018).

### MLST Analysis

Primer sequences and PCR amplification conditions for seven housekeeping genes (*atpD, fusA, glnS, gltB, gyrB, infB*, and *ppsA*) were provided by a previous report (Baldwin et al., 2009). The PCR amplified products were sequenced by Beijing Genomics Institute (BGI, Beijing China). Sequence type (ST) of isolate was determined by comparing it with known 7-loci profiles in the *Cronobacter* PubMLST database (http://www.pubmlst.org/cronobacter). A phylogenetic tree based on seven housekeeping genes was constructed using the Unweighted Pair Group Method with Arithmetic mean (UPGMA) algorithm in MEGA7.0, with 1,000 bootstrap replicates. The *C. sakazakii* ATCC 29544, *C. sakazakii* ATCC BAA-894, *C. sakazakii* ATCC 29004, *C. sakazakii* ATCC 12868, *C. malonaticus* CDC 105877, *C. dublinensis* LMG 23823, *C. turicensis* LMG 23827, *C. universalis* NCTC 9529, *C. condimenti* LMG 26250, and *C. muytjensii* ATCC 51329 were used as reference strains.

### O-Antigen Serotype Analysis

The serotype-specific PCR assays was used to determine the O-antigen serotype (OT) of *Cronobacter* spp. isolates according to previous reports, as follows: *C. sakazakii* serotype (Cs) O1, CsO2, CsO3, CsO4, and CsO7 (Jarvis et al., 2011; Sun et al., 2012b), *C. malonaticus* serotype (Cm) O1 and CmO2 (Blazkova et al., 2015), *C. turicensis* serotype (Ct) O1 to CtO3 (Sun et al., 2012a; Jarvis et al., 2013), and *C. dublinensis* (Cd) O1 and CdO2 (Jarvis et al., 2013). The sequences of 12 pairs of primers used for O-antigen serotype analysis were shown in Supplementary Table 2.

### Biofilm Formation by *Cronobacter* spp. on Tinplate

The biofilm biomass produced by *Cronobacter* spp. on tinplate was measured as described by Fei et al. (2021). In brief, 30 μl of *Cronobacter* pre-culture was inoculated into 24-well polystyrene plates containing 2 ml of TSB. Sterilized tinplate coupons (1 × 1 cm) were inserted vertically into the wells, followed by incubation at 37°C for 48 h to allow the isolates to form biofilms. After three gentle rinses with sterilized distilled water, the cultures on coupons were removed. The biofilms on coupons were stained with 0.1% crystal violet for 30 min, then rinsed to remove the crystal violet, followed by dissolution in 2 ml of 75% ethanol solution. The optical density (OD) value of the above-mentioned solution at wavelengths of 595 nm representing staining intensity was measured to assess the biofilm-forming ability of *Cronobacter* strains. The unincubated strain was used as a control to eliminate the background staining. The above experiments were repeated three times.

### Antibiotic Susceptibility Testing

The Kirby–Bauer disk diffusion method was used to assess the antibiotic susceptibilities of *Cronobacter* isolates according to the guidelines of the Clinical Laboratory Standards Institute (CLSI, 2020). Nine categories of antibiotics were selected for the antibiotic susceptibility testing, β-Lactams: ampicillin (10 μg), amoxicillin (25 μg), cephalothin (30 μg), ampicillin-sulbactam (10:10 μg), cefotaxime (30 μg), and ceftriaxone (30 μg); macrolides: azithromycin (15 μg) and erythromycin (15 μg); sulfonamides: sulfadiazine (250 μg), sulfadoxine (250 μg), and trimethoprim-sulfamethoxazole (1.25/23.75 μg); aminoglycosides: streptomycin (10 μg) and gentamicin (10 μg); amphenicols: chloramphenicol (30 μg); tetracyclines: tetracycline (30 μg) and oxytetracycline (30 μg); glycopeptide: vancomycin (30 μg); fluoroquinolones: ciprofloxacin (5 μg) and pipemidic acid (30 μg); polypeptide: colistin (10 μg). *Escherichia coli* ATCC 25922 was used as the quality control strain. In accordance with the CLSI guidelines, if the diameter of inhibition zone (DIZ) against *Cronobacter* spp. isolates was greater than that of quality control strain, the antibiotic resistance was interpreted as sensitive (S); if it is less than, the antibiotic resistance was defined as resistant (R); if the DIZ was between R and S, the effect of antibiotics was expressed as intermediate (I).

### Statistical Analysis

Biofilm formation test and antibiotic susceptibility test of isolates were carried out in triplicate. The data were presented in the form of mean ± standard deviation, and were statistically analyzed using ANOVA by the SPSS 20.0 software (SPSS Inc., Chicago, IL). *P* ≤ 0.05 was considered significant.

## Results

### Prevalence of *Cronobacter* spp. in Commercial PIF From Nine Provinces in China

As shown in Table 1, the total prevalence rate of *Cronobacter* spp. in PIF from nine provinces in China was 0.75% (27/3600). The highest prevalence rate (1.25%, 5/400) of *Cronobacter* spp. was found in commercial PIF from Shaanxi province, followed by Guizhou (1.00%, 4/400), Yunnan (1.00%, 4/400), Fujian provinces (1.00%, 4/400), Henan (0.75%, 3/400), Jilin (0.50%, 2/400), Hebei (0.50%, 2/400), Xinjiang (0.50%, 2/400), and Heilongjiang (0.25%, 1/400).

**Table 1:** Prevalence of *Cronobacter* spp. in commercial PIF from nine provinces in China.

**Bacterial strains**	**Region**	**No. of** **samples**	**No. of** ***Cronobacter***	**Prevalence** **rate**
C-HLJ1	Heilongjiang	400	1	0.25%
C-J-1, C-J-2	Jilin	400	2	0.50%
C-HB1, C-HB2	Hebei	400	2	0.50%
C-HN1, C-HN2, C-HN3	Henan	400	3	0.75%
C-S-1, C-S-2, C-S-3, C-S-4, C-S-5	Shaanxi	400	5	1.25%
C-G-1, C-G-2, C-G-3, C-G-4	Guizhou	400	4	1.00%
C-Y-1, C-Y-2, C-Y-3, C-Y-4	Yunnan	400	4	1.00%
C-X-1, C-X-2	Xinjiang	400	2	0.50%
C-F-1, C-F-2, C-F-3, C-F-4	Fuzhou	400	4	1.00%
Total		3,600	27	0.75%

### MLST Analysis

**Figure 1:**
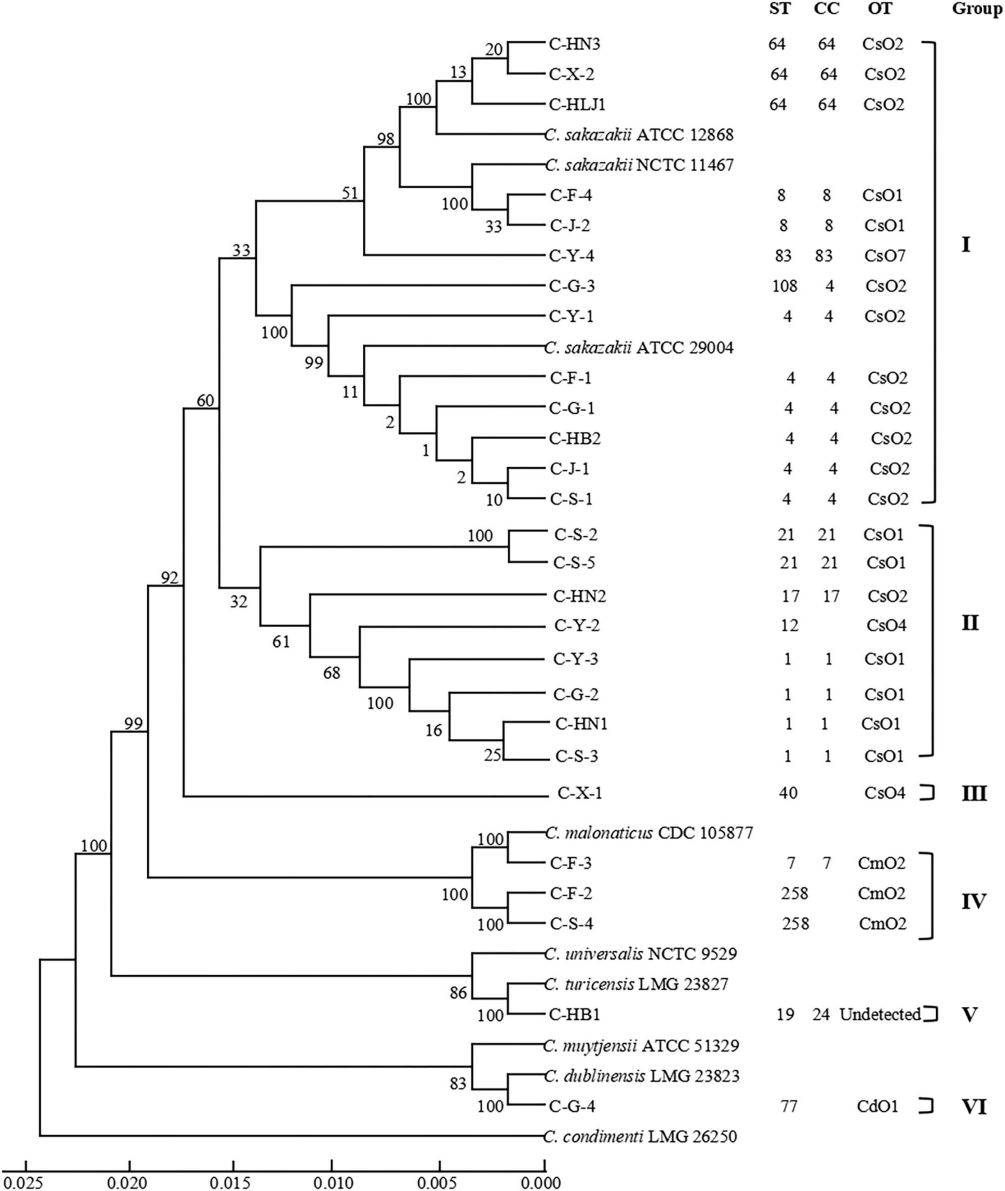
Phylogenetic relationship of 27 *Cronobacter* isolates containing 14 STs. The UPGMA tree based on 7 loci was constructed using MEGA7.0, with 1,000 bootstrap replicates. The *Cronobacter sakazakii* ATCC 29544, *C. sakazakii* ATCC BAA-894, *C. sakazakii* ATCC 29004, *C. sakazakii* ATCC 12868, *Cronobacter malonaticus* CDC 105877, *Cronobacter dublinensis* LMG 23823, *Cronobacter turicensis* LMG 23827, *Cronobacter universalis* NCTC 9529, *Cronobacter condimenti* LMG 26250, and *Cronobacter muytjensii* ATCC 51329 were used as the reference strains. Detailed information about each strain is added, including ST, CC, OT, and group.

As shown in Table 2, 27 *Cronobacter* isolates included 22 *C. sakazakii* strains (81.48%), 3 *C. malonaticus* strains (11.11%), 1 *C. turicensis* strain (3.70%), and 1 *C. dublinensis* strain (3.70%), and were divided into 14 STs using MLST method. Among them, ST4 (6/27, 22.22%), ST1 (4/27, 14.81%), and ST64 (3/27, 11.11%) were the dominant *Cronobacter* STs, meanwhile, clonal complex (CC) 4 (7/27, 25.93%) was the main *Cronobacter* CC. In addition, Figure 1 showed a phylogenetic relationship of 27 isolates based on 3,036 bp-concatenated sequences of 7 housekeeping genes. These isolates were clustered into six groups using 98% similarity as the identification threshold. Group I (13/27, 48.15%) was the dominant group, and contains *C. sakazakii* ST4, ST8, ST64, ST83, and ST108. In addition, *C. sakazakii* ST4 was the most widely distributed, being found in all 6 provinces. However, a clear association between ST and collection region was not found.

**Table 2:** MLST and O-antigen serotyping of 27 *Cronobacter* isolates.

**Strain number**	**Species**	**ID^a^**	**ST**	**CC**	**OT**
C-HLJ1	*C. sakazakii*	3480	64	64	CsO2
C-J-1	*C. sakazakii*	3481	4	4	CsO2
C-J-2	*C. sakazakii*	3482	8	8	CsO1
C-HB1	*C. turicensis*	3483	19	24	Undetected
C-HB2	*C. sakazakii*	3484	4	4	CsO2
C-HN1	*C. sakazakii*	3485	1	1	CsO1
C-HN2	*C. sakazakii*	3486	17	17	CsO2
C-HN3	*C. sakazakii*	3487	64	64	CsO2
C-S-1	*C. sakazakii*	3488	4	4	CsO2
C-S-2	*C. sakazakii*	3489	21	21	CsO1
C-S-3	*C. sakazakii*	3490	1	1	CsO1
C-S-4	*C. malonaticus*	3491	258		CmO2
C-S-5	*C. sakazakii*	3492	21	21	CsO1
C-G-1	*C. sakazakii*	3493	4	4	CsO2
C-G-2	*C. sakazakii*	3494	1	1	CsO1
C-G-3	*C. sakazakii*	3495	108	4	CsO2
C-G-4	*C. dublinensis*	3496	77		CdO1
C-Y-1	*C. sakazakii*	3497	4	4	CsO2
C-Y-2	*C. sakazakii*	3498	12		CsO4
C-Y-3	*C. sakazakii*	3499	1	1	CsO1
C-Y-4	*C. sakazakii*	3500	83	83	CsO7
C-X-1	*C. sakazakii*	3501	40		CsO4
C-X-2	*C. sakazakii*	3502	64	64	CsO2
C-F-1	*C. sakazakii*	3503	4	4	CsO2
C-F-2	*C. malonaticus*	3504	258		CmO2
C-F-3	*C. malonaticus*	3505	7	7	CmO2
C-F-4	*C. sakazakii*	3506	8	8	CsO1

*^a^Strain identification code in the PubMLST Cronobacter database*.

### O-Antigen Serotype Analysis

Table 2 showed that 27 *Cronobacter* strains were classified into 6 OTs. Twenty-two *C. sakazakii* isolates were divided into four *C. sakazakii* serotypes, among them, CsO2 (11/22, 50.00%) was the main OT of *C. sakazakii*, followed by CsO1 (8/22, 36.36%), CsO4 (2/22, 9.09%), and CsO7 (1/22, 4.55%). Three *C. malonaticus* isolates were identified as CmO2. Meanwhile, *C. dublinensis* C-G-4 (ST77) was detected as CdO1, while, the *C. turicensis* serotype of *C. turicensis* C-HB1 (ST19) was not found. Furthermore, the isolates with the same ST were classified into the same OT, however, the isolates with the same OT contained multiple STs, which suggested that the discrimination of O-antigen serotype analysis was lower than that of MLST.

### Ability of Biofilm Formation of *Cronobacter* spp. on Tinplate

**Figure 2:**
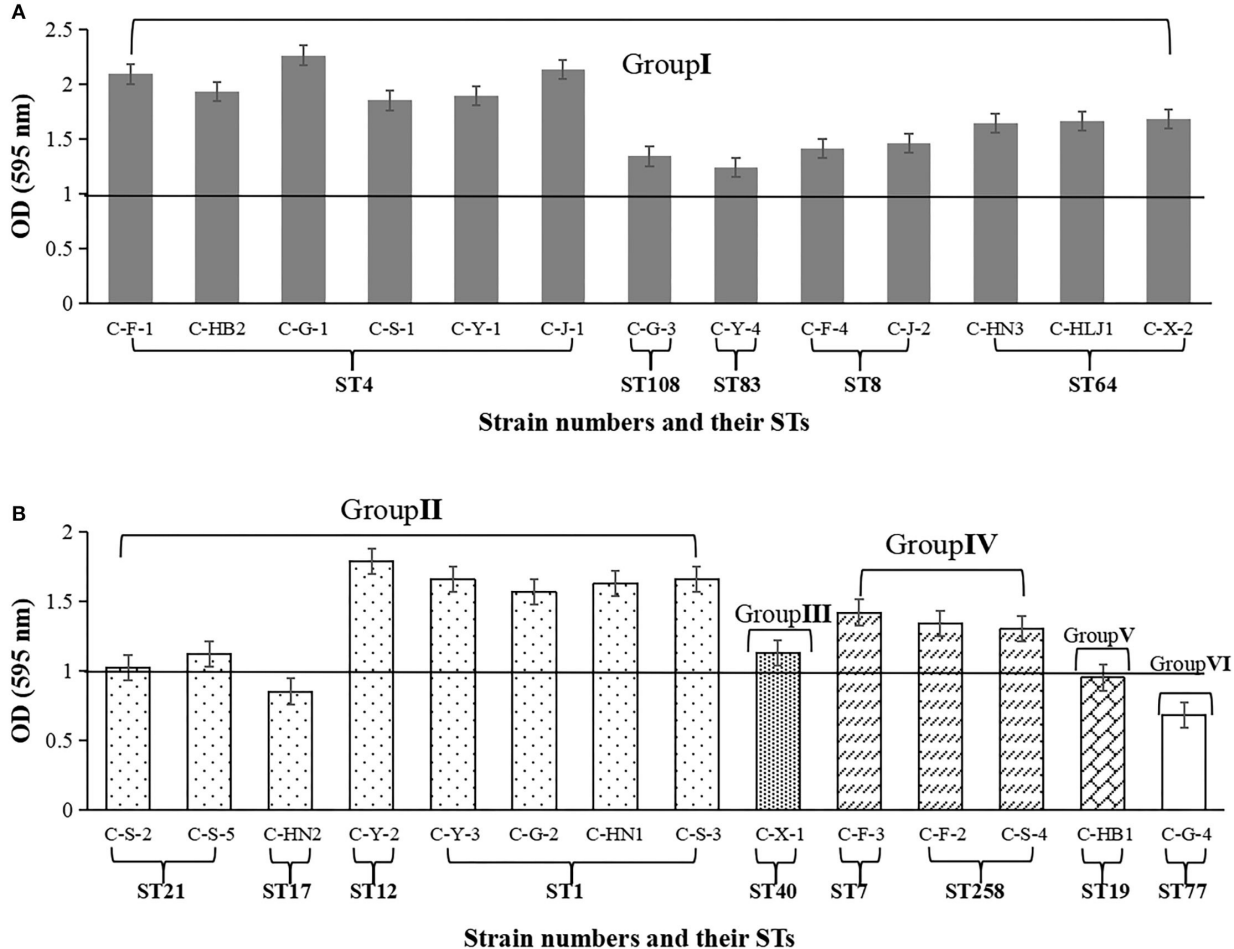
Biofilm biomass formed by 27 *Cronobacter* isolates on tinplate. The OD value at wavelength of 595 nm was used to indicate the biofilm biomass. **(A)** *Cronobacter* isolates belonging to group I. **(B)** *Cronobacter* isolates belonging to group II, group III group IV, group V, and group VI, respectively.

As shown in Figure 2, the biofilm-forming abilities of 27 *Cronobacter* isolates with 14STs on tinplate were tested. Among the 27 *Cronobacter* strains, the OD_595_ values of 24 isolates (88.89%), including 22 strains of *C. sakazakii* and 3 strains of *C. malonaticus*, were >1. Meanwhile, the OD_595_ values of three strains of *C. sakazakii* (C-F-1, C-G-1, and C-J-1) were >2, while the OD_595_ values of the three strains (*C. sakazakii* C-HN2, *C. dublinensis* C-HB1, and *C. turicensis* C-G-4) were <1. In addition, there was a correlation between the ST and biofilm-forming ability of *Cronobacter* strains. The ability to form biofilm of strains with ST4 (OD_595_ = 2.03 ± 0.14) was significantly stronger than that of strains with other STs (*P* < 0.05), followed by *C. sakazakii* ST12 (OD_595_ = 1.78 ± 0.13), *C. sakazakii* ST64 (OD_595_ = 1.66 ± 0.13), *C. sakazakii* ST1 (OD_595_ = 1.63 ± 0.33), and *C. malonaticus* ST258 (OD_595_ = 1.32 ± 0.16).

### Antibiotic Susceptibility Analysis

Table 3 showed the antibiotic susceptibilities of 27 *Cronobacter* isolates to 20 antibiotics (9 categories). All isolates were susceptible to ampicillin-sulbactam, ceftriaxone, cefotaxime, sulfadiazine, sulfadoxine, trimethoprim-sulfamethoxazole, gentamicin, tetracycline, ciprofloxacin, and colistin. The majority of isolates were susceptible to oxytetracycline (96.30%), pipemidic acid (96.30%), azithromycin (92.59%), erythromycin (88.89%), streptomycin (88.89%), amoxicillin (85.19%), and ampicillin (81.48%). While, 55.56 and 96.30% of strains were resistant to cephalothin and vancomycin, respectively. In addition, *C. sakazakii* C-Y-4 (ST83), C-S-2(ST21), and C-S-5(ST21) exhibited resistance to three antibiotics (ampicillin, cephalothin, and vancomycin).

**Table 3:** Antibiotic susceptibility of 27 *Cronobacter* isolates.

**Category**	**Antimicrobial agent**	***Cronobacter*** **isolates (*****n*** **=** **27)**		
		**No. (%) of S**	**No. (%) of I**	**No. (%) of R**
β-Lactams	Ampicillin	22 (81.48)	0 (0.00)	5 (18.52)
	Amoxicillin	23 (85.19)	1 (3.70)	3 (11.11)
	Cephalothin	2 (7.41)	10 (37.04)	15 (55.56)
	Ampicillin-sulbactam	27 (100.00)	0 (0.00)	0 (0.00)
	Ceftriaxone	27 (100.00)	0 (0.00)	0 (0.00)
	Cefotaxime	27 (100.00)	0 (0.00)	0 (0.00)
Macrolides	Azithromycin	25 (92.59)	0 (0.00)	2 (7.41)
	Erythromycin	24 (88.89)	1 (3.70)	2 (7.41)
Sulfonamides	Sulfadiazine	27 (100.00)	0 (0.00)	0 (0.00)
	Sulfadoxine	27 (100.00)	0 (0.00)	0 (0.00)
	Trimethoprim-sulfamethoxazole	27 (100.00)	0 (0.00)	0 (0.00)
Aminoglycosides	Streptomycin	24 (88.89)	2 (7.41)	1 (3.70)
	Gentamicin	27 (100.00)	0 (0.00)	0 (0.00)
Amphenicols	Chloramphenicol	20 (74.07)	5 (18.52)	2 (7.41)
Tetracyclines	Tetracycline	27 (100.00)	0 (0.00)	0 (0.00)
	Oxytetracycline	26 (96.30)	0 (0.00)	1 (3.70)
Glycopeptide	Vancomycin	1 (3.70)	0 (0.00)	26 (96.30)
Fluoroquinolones	Ciprofloxacin	27 (100.00)	0 (0.00)	0 (0.00)
	Pipemidic acid	26 (96.30)	0 (0.00)	1 (3.70)
Polypeptide	Colistin	27 (100.00)	0 (0.00)	0 (0.00)

## Discussion

Over the years, many studies have been devoted to investigating the occurrence of *Cronobacter* spp. in PIF in the Chinese market, in order to provide an early warning of risk to consumers and public safety (Pan et al., 2014; Fei et al., 2017b; Zhang et al., 2017; Ling et al., 2021). Pan et al. (2014) detected 19 *Cronobacter* strains from 165 PIF samples, with a prevalence rate of 11.50%, from December 2011 to December 2012. Zhang et al. (2017) found that from 2011 to 2013, the prevalence rates of *Cronobacter* spp. in commercial PIF was 1.60% in Shandong, China. Further, Fei et al. (2017a,b) investigated the presence of *Cronobacter* spp. in PIF collected from Chinese retail markets from January 2015 to March 2017, and the prevalence rate was 2.80%. Ling et al. (2021) reviewed the incidences (Range from 0.90 to 23.10%) of *Cronobacter* spp. isolated from PIF in China from 2004 to 2018, which claimed to cover over 10,000 PIF samples in total. Compared with the above studies, in this study, the prevalence rate (0.75%) of *Cronobacter* spp. in commercial PIF was obviously reduced, indicating that these continuous studies have attracted the attention of government departments and manufacturers and contributed to the improvement of *Cronobacter* spp. control measures. It should be emphasized that almost all the methods for the isolation of *Cronobacter* spp. from China, including this study, were carried out according to the standard of China GB4789.40-2010 (Pan et al., 2014; Fei et al., 2017b; Zhang et al., 2017; Li et al., 2019; Li C. et al., 2020; Zeng et al., 2020). According to this national standard, the mLST-Vm medium was used for selective enrichment of bacteria solution, and its culture temperature was 44°C, which may lead to the failure of some *Cronobacter* strains to grow (International Standards Organization [ISO], 2017). On the other hand, the raw milk of PIF collected from Shaanxi province was goat milk, and the prevalence rate of this pathogen in these samples is the highest, whether there is a correlation between the two needs further study. In addition, we found that the prevalence rate of *Cronobacter* spp. was related to the distance between the place of production (Inner Mongolia) and the place of sale (except Shaanxi), and the farther the distance was, the higher the prevalence rate was (Supplementary Figure 1), which suggests that we need to strengthen the hygiene management in the PIF logistics process.

In this study, 27 *Cronobacter* strains isolated from commercial PIF contained four species, and were divided into 14 STs. Previous studies have shown that *C. sakazakii, C. malonaticus*, and *C. turicensis* are associated with the neonatal infections in seven species of *Cronobacter* spp. (Forsythe, 2005; Joseph et al., 2012), and the strains belonging to the above 3 species were detected in our study. Meanwhile, among the 14 STs, several important STs have been found. The details are as follows: *C. sakazakii* ST4 is the main ST leading to neonatal meningitis (Joseph et al., 2012), *C. sakazakii* ST1 is the dominant ST isolated from PIF (Sonbol et al., 2013), *C. sakazakii* ST12 is associated with infant necrotizing enterocolitis (Forsythe et al., 2014), and *C. sakazakii* ST8 is mainly isolated from clinical cases (Sonbol et al., 2013). In addition, *C. sakazakii* ST4, ST1, and ST64 were the dominant STs isolated from PIF in this study, which was consistent with previous studies (Fei et al., 2015, 2017b). But, ST64 was not the predominant ST of *Cronobacter* strains from PIF in other countries (Sonbol et al., 2013). Interestingly, *C. sakazakii* ST64 was also the dominant ST isolated from Chinese aquatic products, meat, and meat products (Li C. et al., 2020; Zeng et al., 2020). Therefore, *C. sakazakii* strains with ST64 should be heavily prevented and controlled for China's food industry.

As a supplement to MLST, the O-antigen serotype analysis was performed to reveal information related to the pathogenicity of *Cronobacter* isolates. The predominant serotype of *Cronobacter* isolates from commercial PIF in the current study was CsO2, which was closely correlated with the *C. sakazakii* ST4 causing neonatal meningitis. As comparisons, the main serotype of *Cronobacter* strains in aquatic products and edible mushrooms was CsO1 (Li et al., 2019; Li C. et al., 2020), as well as, CsO1 and CsO2 were the dominant serotypes of *Cronobacter* spp. in the spices, cereals, meat, and meat products (Li et al., 2017; Zeng et al., 2020). In addition, CsO3 was found in aquatic products, edible mushrooms, meat, and meat products (Li et al., 2019; Li C. et al., 2020; Zeng et al., 2020), but this serotype was not detected in our study.

Previous studies mainly focus on the biofilm formation of *Cronobacter* strains on stainless steel, however, most of this pathogen in PIF comes from re-contamination during packaging and logistics (Lee et al., 2012; Fei et al., 2015; Huang et al., 2020). Therefore, this study evaluated the biofilm-forming ability of *Cronobacter* isolates on tinplate that is the commonly used packaging material. In this study, we found that there was a certain relationship between the biofilm biomass and ST of *Cronobacter* spp., and the strains with ST4 had a higher ability to produce biofilm, which may be one reason why *C. sakazakii* ST4 is so prevalent in the environment. In addition, Gupta et al. (2018) revealed the ability of *C. sakazakii* to form biofilms on different materials, including stainless steel, polyurethane, polyvinyl chloride, and silicone, and found that polyvinyl chloride and silicone were the higher risk materials.

In order to better select effective antibiotics, 20 antibiotics belonging to 9 categories were used to test the antibiotic susceptibility of *Cronobacter* isolated from commercial PIF. In this study, all isolates were susceptible to all sulfonamides antibiotics, while most isolates were resistant to cephalothin and vancomycin, which showed similar resistance to most antibiotics as in previous studies (Fei et al., 2017b; Li et al., 2019; Li C. et al., 2020). Differently, compared with *Cronobacter* strains isolated from aquatic and meat products, the isolates in this study were more resistant to ampicillin and more susceptible to cephalothin and trimethoprim/sulfameth-oxazol (Li C. et al., 2020; Zeng et al., 2020). In addition, in this study, three isolates (11.11%) from PIF samples were found to be resistant to three antibiotics, which suggests that with the long-term use of antibiotics, multidrug-resistant strains are emerging, and that monitoring of antibiotic susceptibility of isolates should be continuous for early warning purposes.

## Conclusions

In conclusion, we revealed the prevalence, genotype, biofilm formation, and antibiotic susceptibility of *Cronobacter* spp. in commercial PIF collected from nine provinces in China. Compared with before, a lower prevalence rate of *Cronobacter* spp. in PIF was found. *C. sakazakii* ST4, ST1, and ST64 were still the dominant STs in commercial PIF, and the isolates belonging to dominant STs had a stronger ability to produce biofilms. Sulfonamides were recommended for the treatment of *Cronobacter* infection. Our findings help to reveal the contamination status and characterization of *Cronobacter* spp. in PIF from Chinese markets, and provide the guidance for effectively preventing the contamination of this pathogen in PIF.

## Data Availability Statement

The data presented in the study are deposited in the Cronobacter PubMLST database repository (http://www.pubmlst.org/cronobacter), accession number 3480-3506.

## Author Contributions

PF, WY, YC, and ZM conceived and designed the experiments. PF, HJ, YM, GD, SL, XC, SJ, QX, and WY performed the experiments. PF, YC, and WY supervised the project. PF, XC, and YC analyzed the data. PF wrote the paper. All authors contributed to the article and approved the submitted version.

## Funding

This research was supported by the Natural Science Foundation of Henan Province (212300410137), Natural Science Foundation of Guizhou Province (Qiankehe Foundation-ZK [2022] General 008), Key Scientific Research Projects of Institutions of Higher Learning of Henan (21A550007), Liaoning Provincial Science and Technology Department issued the Key Research and Development Project of Tackling Key Problems and Industrialization (2017225065), and Innovation and Entrepreneurship Training Program of College Students of Henan Province (202110464027).

## Conflict of Interest

ZM is employed by Inner Mongolia Meng Niu Dairy Industry Group Co., Ltd. SJ and QX are employed by Heilongjiang Feihe Dairy Co., Ltd. The remaining authors declare that the research was conducted in the absence of any commercial or financial relationships that could be construed as a potential conflict of interest.

## Publisher's Note

All claims expressed in this article are solely those of the authors and do not necessarily represent those of their affiliated organizations, or those of the publisher, the editors and the reviewers. Any product that may be evaluated in this article, or claim that may be made by its manufacturer, is not guaranteed or endorsed by the publisher.

## Supplementary Material

Supplementary Figure 1 Sampling sites and milk source base of commercial PIF used in this study.

Supplementary Table 1 The specific information of all samples.

Supplementary Table 2 The sequences of 12 pairs of primers used for O-antigen serotype analysis.
