# Supplementary figures and images for: Cronobacter spp. in Commercial Powdered Infant Formula Collected From Nine Provinces in China: Prevalence, Genotype, Biofilm Formation, and Antibiotic Susceptibility
Source: Front Microbiol. 2022 May 27;13:900690. doi: 10.3389/fmicb.2022.900690 (PMC9197194; doi:10.3389/fmicb.2022.900690)

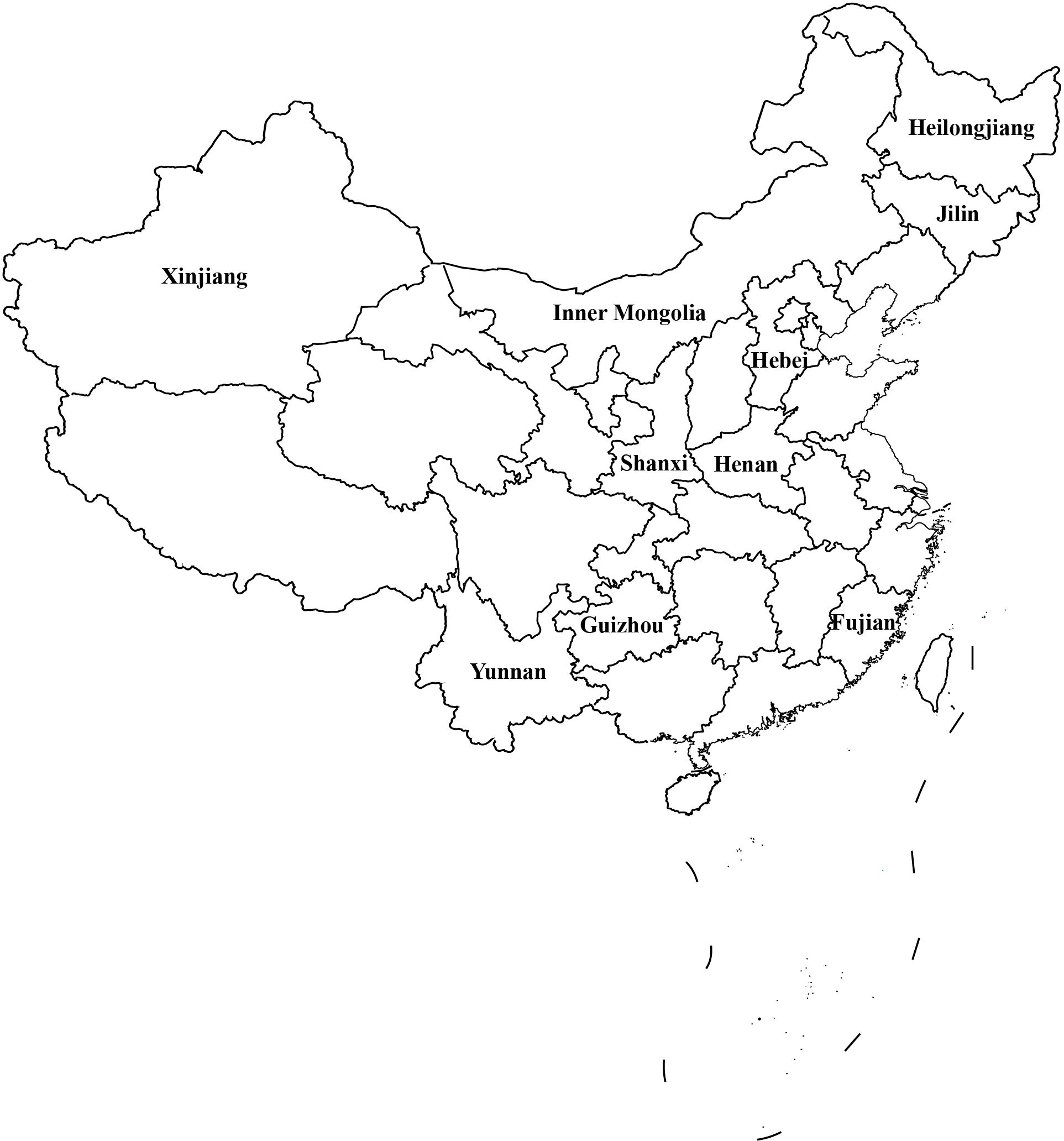

Supplement: Supplementary Figure 1 — Sampling sites and milk source base of commercial PIF used in this study. [file Image_1.JPEG]
