# Supplementary material for: Cronobacter spp. in Commercial Powdered Infant Formula Collected From Nine Provinces in China: Prevalence, Genotype, Biofilm Formation, and Antibiotic Susceptibility
Source: Front Microbiol. 2022 May 27;13:900690. doi: 10.3389/fmicb.2022.900690 (PMC9197194; doi:10.3389/fmicb.2022.900690)
Supplement: Supplementary Table 1 — The specific information of all samples. [file Table_1.doc]

**Supplementary Table 1** The specific information of all samples.

| Province | City | S[ample](javascript:;) [number](javascript:;) | [Collection](javascript:;) [time](javascript:;) | Manufacturer | [Place](javascript:;) [of](javascript:;) [origin](javascript:;) | Raw milk type |
| --- | --- | --- | --- | --- | --- | --- |
| Heilongjiang | Harbin | 50 | June 2019 | a, b, c | A, B, C, | Cow milk |
|  |  | 50 | July 2020 | a, b, c |  | Cow milk |
|  | [Kiamusze](javascript:;) | 50 | June 2019 | a, b, c |  | Cow milk |
|  |  | 50 | July 2020 | a, b, c |  | Cow milk |
|  | Andan | 50 | June 2019 | a, b, c |  | Cow milk |
|  |  | 50 | July 2020 | a, b, c |  | Cow milk |
|  | [Mudanjiang](javascript:;) | 50 | June 2019 | a, b, c |  | Cow milk |
|  |  | 50 | July 2020 | a, b, c |  | Cow milk |
| Jilin | Changchun | 50 | August 2018 | a, b, c | A, D, E | Cow milk |
|  |  | 50 | October 2019 | a, b, c |  | Cow milk |
|  | Jilin | 50 | August 2018 | a, b, c |  | Cow milk |
|  |  | 50 | October 2019 | a, b, c |  | Cow milk |
|  | Siping | 50 | August 2018 | a, b, c |  | Cow milk |
|  |  | 50 | October 2019 | a, b, c |  | Cow milk |
|  | Baishan | 50 | August 2018 | a, b, c |  | Cow milk |
|  |  | 50 | October 2019 | a, b, c |  | Cow milk |
| Hebei | Tangshan | 50 | March 2018 | a, b, c | A, B, C, F, G, | Cow milk |
|  |  | 50 | April 2020 | a, b, c |  | Cow milk |
|  | [Langfang](javascript:;) | 50 | March 2018 | a, b, c |  | Cow milk |
|  |  | 50 | April 2020 | a, b, c |  | Cow milk |
|  | [Shijiazhuang](javascript:;) | 50 | March 2018 | a, b, c |  | Cow milk |
|  |  | 50 | April 2020 | a, b, c |  | Cow milk |
|  | [Qinhuangdao](javascript:;) | 50 | March 2018 | a, b, c |  | Cow milk |
|  |  | 50 | April 2020 | a, b, c |  | Cow milk |
| Henan | Zhengzhou | 50 | March 2018 | a, b, c | H, I, J, K, A, | Cow milk |
|  |  | 50 | June 2019 | a, b, c |  | Cow milk |
|  | Luoyang | 50 | March 2019 | a, b, c |  | Cow milk |
|  |  | 50 | June 2019 | a, b, c |  | Cow milk |
|  | Nanyang | 50 | May 2020 | a, b, c |  | Cow milk |
|  |  | 50 | August 2020 | a, b, c |  | Cow milk |
|  | Jiyuan | 50 | May 2020 | a, b, c |  | Cow milk |
|  |  | 50 | August 2020 | a, b, c |  | Cow milk |
| Guizhou | Guiyang | 50 | March 2018 | a, b, c | L, M, N, A, E | Cow milk |
|  |  | 50 | February 2019 | a, b, c |  | Cow milk |
|  | Bijie | 50 | March 2018 | a, b, c |  | Cow milk |
|  |  | 50 | February 2019 | a, b, c |  | Cow milk |
|  | Zunyi | 50 | March 2018 | a, b, c |  | Cow milk |
|  |  | 50 | April 2020 | a, b, c |  | Cow milk |
|  | Liupanshui | 50 | March 2018 | a, b, c |  | Cow milk |
|  |  | 50 | April 2020 | a, b, c |  | Cow milk |
| Xinjiang | [Urumchi](javascript:;) | 100 | February 2019 | a, b, c | A, B, D, O | Cow milk |
|  |  | 100 | September 2020 | a, b, c |  | Cow milk |
|  | [Turpan](javascript:;) | 50 | February 2019 | a, b, c |  | Cow milk |
|  |  | 50 | September 2020 | a, b, c |  | Cow milk |
|  | [Karamay](javascript:;) | 50 | February 2019 | a, b, c |  | Cow milk |
|  |  | 50 | September 2020 | a, b, c |  | Cow milk |
| Yunnan | Kunming | 100 | February 2020 | a, b, c | M, N, A, D | Cow milk |
|  |  | 100 | August 2020 | a, b, c |  | Cow milk |
|  | Zhaotong | 50 | February 2020 | a, b, c |  | Cow milk |
|  |  | 50 | August 2020 | a, b, c |  | Cow milk |
|  | Yuxi | 50 | February 2020 | a, b, c |  | Cow milk |
|  |  | 50 | August 2020 | a, b, c |  | Cow milk |
| Fujian | Fuzhou | 50 | December 2018 | a, b, c | A, B, K, E | Cow milk |
|  |  | 50 | October 2019 | a, b, c |  | Cow milk |
|  | Putian | 50 | December 2018 | a, b, c |  | Cow milk |
|  |  | 50 | May 2020 | a, b, c |  | Cow milk |
|  | Quanzhou | 50 | December 2018 | a, b, c |  | Cow milk |
|  |  | 50 | May 2020 | a, b, c |  | Cow milk |
|  | Xiamen | 50 | December 2018 | a, b, c |  | Cow milk |
|  |  | 50 | October 2019 | a, b, c |  | Cow milk |
| Shaanxi | Xi’an | 50 | March 2018 | a, b, c, d | H, I, G, P | Goat milk |
|  |  | 50 | December 2018 | a, b, c, d |  | Goat milk |
|  | Hanzhong | 50 | March 2018 | a, b, c, d |  | Goat milk |
|  |  | 50 | December 2018 | a, b, c, d |  | Goat milk |
|  | Weinan | 50 | March 2018 | a, b, c, d |  | Goat milk |
|  |  | 50 | December 2018 | a, b, c, d |  | Goat milk |
|  | Xianyang | 50 | March 2018 | a, b, c, d |  | Goat milk |
|  |  | 50 | December 2018 | a, b, c, d |  | Goat milk |

Different lowercase letters indicate different different manufacturers.

Different capital letters indicate different [place](javascript:;) [of](javascript:;) [origin](javascript:;)s.
