# Supplementary material for: Cronobacter spp. in Commercial Powdered Infant Formula Collected From Nine Provinces in China: Prevalence, Genotype, Biofilm Formation, and Antibiotic Susceptibility
Source: Front Microbiol. 2022 May 27;13:900690. doi: 10.3389/fmicb.2022.900690 (PMC9197194; doi:10.3389/fmicb.2022.900690)
Supplement: Supplementary Table 2 — The sequences of 12 pairs of primers used for O-antigen serotype analysis. [file Table_2.doc]

**Supplementary Table 2** The sequences of 12 pairs of primers used for O-antigen serotype analysis

| *Cronobacter* spp. | Primer | Sequence (5′ to 3′) |
| --- | --- | --- |
| *C. sakazakii* | CsO1 | CCCGCTTGTATGGATGTT |
|  |  | CTTTGGGAGCGTTAGGTT |
|  | CsO2 | ATTGTTTGCGATGGTGAG |
|  |  | AAAACAATCCAGCAGCAA |
|  | CsO3 | CTCTGTTACTCTCCATAGTGTTC |
|  |  | GATTAGACCACCATAGCCA |
|  | CsO4 | ACTATGGTTTGGCTATACTCCT |
|  |  | ATTCATATCCTGCGTGGC |
|  | CsO7 | CATTTCCAGATTATTACCTTTC |
|  |  | ACACTGGCGATTCTACCC |
| *C. malonaticus* | CmO1 | CCTTCTTCATTAGCCATTG |
|  |  | CAGACGACTTACGCCTTG |
|  | CmO2 | TGGCCCTTGTTAGCAAGACGTTTC |
|  |  | ATCCACATGCCGTCCTTCATCTGT |
| *C. turicensis* | CtO1 | TACCCACTCCTCCAAGAACG |
|  |  | TTTGTCACGAGAGCGGTTGAATAC |
|  | CtO2 | TTTCTTGTTATTGCCTGTGT |
|  |  | AACAAAATCAGCGAGACTAA |
|  | CtO3 | GCATCCCTTCAGAGTAGCGCA |
|  |  | ACCACCTGCCATTGTCCTACTG |
| *C. dublinensis* | CdO1 | ACACCTCCTACGAACTTGAAGC |
|  |  | TAAGGTAGCTGCTCAGCTCG |
|  | CdO2 | TTGTCGCTGCGGGTATCAAAG |
|  |  | ACGCACTTTCTCGATGAACTCG |
